# Deregulation of microRNAs by HIV-1 Vpr leads to the development of neurocognitive disorders

**DOI:** 10.1186/1742-4690-8-S2-P51

**Published:** 2011-10-03

**Authors:** Ruma Mukerjee, J Robert Chang, Luis Del Valle, Asen Bagashev1, Monika M Gayed, Randolph B Lyde, Brian J Hawkins, Eric Cohen, Chris Power, S Ausim Azizi, Bassel E Sawaya

**Affiliations:** 1 Molecular studies of Neurodegenerative Diseases Lab, Department of Neurology Temple University School of Medicine, Philadelphia, PA 19140, US; 2 Department of Medicine [Section of Hematology/Oncology] & Department of Pathology Louisiana State University School of Medicine, Stanley S. Scott Cancer Center, CSRB Suite 524, New Orleans, LA 70112, US; 3 Anesthesiology and Pain Medicine, Mitochondria and Metabolism Center, University of Washington, Seattle, WA 98109, US; 4 lnstítut de Recherches Clinigues de Montréal (IRCM) and Department of Microbiology and Immunology, Université de Montréal, Quebec, Canada; 5 Departments of Medicine (Neurology), Medical Microbiology & Immunology and Psychiatry; 6-11 Heritage Medical Research Centre, University of Alberta, Edmonton, Alberta, Canada

## 

Studies have shown that HIV-infected patients develop neurocognitive disorders that are marked by neuronal dysfunction. The lack of productive infection of neurons by HIV suggests that viral and cellular proteins, with neurotoxic activities, released from HIV-1 infected target cells cause this neuronal deregulation. The viral protein R, a protein encoded by HIV-1, has been shown to alter the expression of various important cytokines and inflammatory proteins in infected and uninfected cells, however the mechanisms involved remain unclear. Using human neuronal cell line, we found that Vpr can be taken up by neurons causing *i*- deregulation of calcium homeostasis, *ii*- endoplasmic reticulum-calcium release, *iii*-activation of the oxidative stress pathway, *iv*- mitochondrial dysfunction and *v-* synaptic retraction. In search for the cellular factors involved, we performed microRNAs and gene array assays using human neurons (primary cultures or cell line, SH-SY5Y) that we treated with recombinant Vpr proteins. Interestingly, Vpr deregulates the levels of several miRNAs (e.g. miR-34a) and their target genes (e.g. CREB), which could lead to neuronal dysfunctions. Therefore, we conclude that Vpr is a major player in neuronal dysfunction through deregulating miRNAs and their target genes, a phenomenon that could lead to the development of HAND.

